# A suspended layer additive manufacturing approach to the bioprinting of tri-layered skin equivalents

**DOI:** 10.1063/5.0061361

**Published:** 2021-11-30

**Authors:** Richard J. A. Moakes, Jessica J. Senior, Thomas E. Robinson, Miruna Chipara, Aleksandar Atanasov, Amy Naylor, Anthony D. Metcalfe, Alan M. Smith, Liam M. Grover

**Affiliations:** 1 School of Chemical Engineering, University of Birmingham, Birmingham B15 2TT, United Kingdom; 2 Department of Pharmacy, University of Huddersfield, Queensgate, Huddersfield HD1 3DH, United Kingdom; 3 Inflammation and Ageing, University of Birmingham, Birmingham B15 2TT, United Kingdom

## Abstract

Skin exhibits a complex structure consisting of three predominant layers (epidermis, dermis, and hypodermis). Extensive trauma may result in the loss of these structures and poor repair, in the longer term, forming scarred tissue and associated reduction in function. Although a number of skin replacements exist, there have been no solutions that recapitulate the chemical, mechanical, and biological roles that exist within native skin. This study reports the use of suspended layer additive manufacturing to produce a continuous tri-layered implant, which closely resembles human skin. Through careful control of the bioink composition, gradients (chemical and cellular) were formed throughout the printed construct. Culture of the model demonstrated that over 21 days, the cellular components played a key role in remodeling the supporting matrix into architectures comparable with those of healthy skin. Indeed, it has been demonstrated that even at seven days post-implantation, the integration of the implant had occurred, with mobilization of the adipose tissue from the surrounding tissue into the construct itself. As such, it is believed that these implants can facilitate healing, commencing from the fascia, up toward the skin surface—a mechanism recently shown to be key within deep wounds.

## INTRODUCTION

Chronic wounds that arise following trauma, surgery, or disease pose a major healthcare problem, with around 2.2 × 10^6^ people consequently requiring treatment in the UK alone.^1^ Such wounds are a result of an imbalance within the healing equilibrium, often caused by infection or over production of cytokines within the wound, and ultimately preventing the wound from exiting the inflammatory stage of healing.^2,3^ Consequently, as frequently observed in ulceration, this can often drive necrosis of the underlying tissue, forming deep wounds.^4–6^ Thus, depth is an important consideration, posing a significant challenge to treatments.^7^ To date, the current gold standard in repairing chronic wounds is a split-thickness autograft. However, the donor site is not always of sufficient in thickness to compensate for the extent of tissue damage. Additionally, the technique also faces many other challenges, including morbidity, rejection, and strains on donor banks and tissue demand.^8^

The potential impact of a replacement for skin autografts that are able to enhance wound closure is clear. However, one of the toughest challenges faced when fabricating autograft replacements is replicating the complex skin architectures, both on a micro- and macro-scale.^9,10^ Skin can be typically categorized into three main layers:^11^ the epidermis, with its primary role to provide a barrier to the external environment; dermis, which provides the structural support and elasticity as a connective tissue between the epidermis and underlying tissues; and the hypodermis, again providing support, protection, and temperature control alongside a means of energy storage and mass transport of nutrients, through vessels and ducts alongside neurones and hair follicles. The function of these layers is driven through their physico-chemical properties, dictating the microstructures of the tissues and, from a mechanical point of view, their bulk properties. More recently, the significant role that hypodermis plays within wound closure/healing has become clearer, suggesting that healing progresses upward through the fascia.^12^ As such, adipose-derived mesenchymal stem cells (ADSCs) may contribute to epidermal morphogenesis, neovascularization, and acceleration of wound healing.^13–18^ The true, complex, hierarchical nature of native skin is, therefore, seldom recapitulated in dermo-epidermal constructs alone.^19^

The quality and complexity of skin models and substitutes has significantly progressed over recent years, with the promise of addressing the physical, psychological, and economic issues that are associated with large-scale tissue loss.^20^ Typically, when assessing the literature on these advances, it is possible to categorize the work into either a biological or materials science approach. The latter has progressed rapidly based on understanding materials such as decellularized tissues, modern synthetic polymers, and hydrogels, coupled with new fabrication techniques, such as electrospinning and 3D printing.^9,21–23^ Although these analogues exhibit the desired mechanical behaviors, they do not provide the biological stimulus that is required to accelerate wound closure and regenerate the native environment. In response, biologically driven models rely on using cells as the fundamental building blocks for the constructs. Efforts over the past decade have modeled different aspects of skin, such as its appendages (hair follicles, glands, etc.)^24^ as well as skin-on-a-chip technologies to screen native functions: permeability and cell response to actives.^25,26^ Various models have been proposed ranging upward from mono-cell cultures, with three-layered organoids providing the most reliable platforms for studying tissue function and response.^27,28^ However, achieving physiologically relevant thicknesses is often problematic, particularly in self-assembled models, where cells are seeded in an overlapping manner. Typically, these structures are only ca. 100–200 *μ*m in depth, while native skin's thickness is on the millimeter scale.^24,29^ To this end, cells have been integrated into scaffolds, whereby compartmentalization of cell types (keratinocyte, dermal fibroblasts, and ADSC) within the construct attempts to simulate native structures. Unfortunately, such models still fall short of replicating the microenvironments surrounding the cellular entities, with uniform scaffolds failing to provide the chemical and mechanical gradients throughout the epidermis, dermis, and hypodermis.^30,31^

The use of additive layer manufacturing techniques has also recently attracted significant attention for manufacturing tissue analogues, both hard and soft.^32^ Its ability to produce heterogeneous structures within a single monolith could be used to recapitulate the complexity of human skin. Novel techniques such as melt electro-spin writing (MEW) have allowed the tailoring of mechanical properties by manipulating the patterning of the print.^33^ Such control over the print at nano-length scales provides constructs with the inherent ability to undergo repeatable large deformations across a singular axis.^34^ Furthermore, using a combinatorial approach and co-extruding with a simple hydrogel, it is possible to impregnate such support matrices with defined cell types and boundaries.^35^ Unfortunately, many of the additive layer manufacturing techniques are limited by the types of bioinks available, and/or the balance between printing resolution/fidelity and overall size of the construct. To date, many bioinks are either restricted to high viscosities, needing to be able to self-support throughout the curing processes (temperature-driven transitions, light-based curing, and addition of crosslinkers). This significantly narrows the library of usable materials, often focusing on synthetic polymers, which lack the key chemical compositions of native tissues. Furthermore, complex geometries within the print are often hindered by the need to provide supporting materials (bridges and forks), requiring post-printing steps to remove the excess material. Again, this adds an extra layer of complexity, with support structures requiring chemistries to which the main structure is inert, in order to remove them—providing further challenges to compatibility with biologics, cells, and the route through to clinic.

Suspended layer additive manufacture is a method that provides greater freedom in regard to bioinks, print complexity, and construct size.^36–38^ The method takes advantage of a suspension reservoir, which provides the support for each printed layer. Typically, the supporting material requires specific rheological properties, including shear thinning, and rapid recovery on returning to a zero-shear environment. Such behaviors have been demonstrated by fluid gels, a suspension of micro-gelled particles that reversibly form a weak network in the absence of shear.^39^ To this end, they are easily deformed as the print head moves through them, rapidly recovering a network around the deposited ink and holding it in place throughout curing. Although to date not investigated at the microscale, long range diffusion of the inks printed using this technique appears hindered, remaining localized within the print area, allowing fabrication of complex geometries.^40^ As such, the materials employed within the bioinks are not limited to exhibiting high viscosities/rapid curing in order to prevent collapse of the structure following extrusion, but, in fact, can reflect the true mechanics of the tissue being replicated. Therefore, selection can be based on compatibility with the native extracellular matrices being replicated, as opposed to enabling print conduciveness.^36,40^

This study reports the use of a suspended layer additive manufacturing method to produce cell containing layered constructs with structural and mechanical similarities to human skin. This method has previously been used to manufacture complex and heterogenous tissue-like structures, while maintaining the phenotype of the entrapped cells.^39,40^ The capacity of the manufacturing method to produce a layered structure was assessed, along with its ability to support and maintain cell viability. Furthermore, the migration of cells from the construct into harvested porcine skin was evaluated over a period of 14 days post-incubation.

## RESULTS AND DISCUSSION

**FIG. 1.**
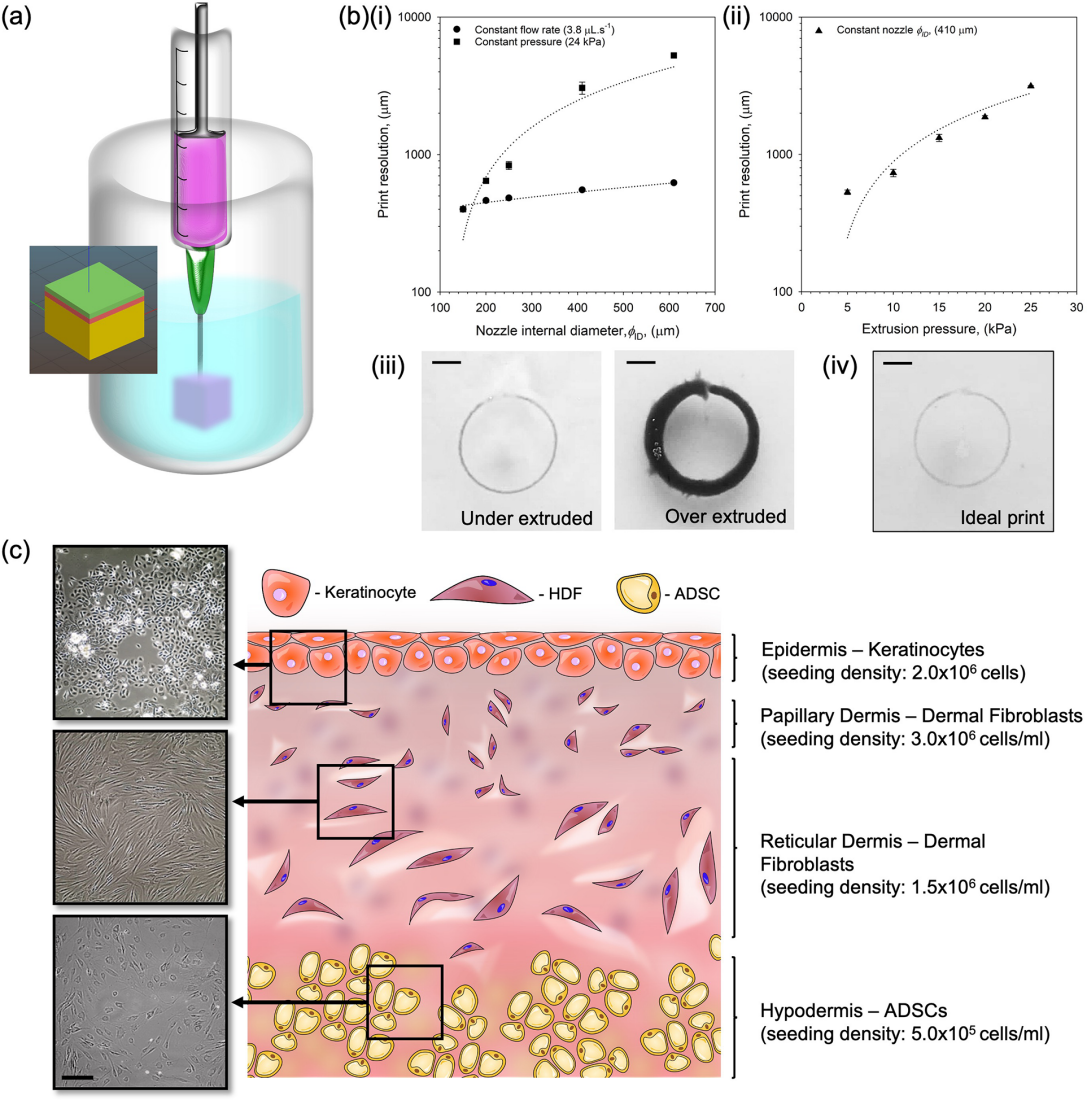
Design and optimization of the implantable construct: (a) diagram of the printing process showing the print suspended in a print bath containing fluid gel (inset shows the CAD image used to print the three-layered construct). (b) Print resolution data obtained for a single layer while controlling various printing parameters: (i) constant flow rate (y = 0.4x + 362; R^2^ = 0.952) and extrusion pressure (y = 8.9x − 1099; R^2^ = 0.985) and (ii) constant nozzle internal diameter (ϕ_ID_) (y = 107.6x − 116; R^2^ = 0.926) (Scale bar = 5 mm). (iii) Exemplar images demonstrating layers that were printed using a ϕ_ID_ 610 *μ*m nozzle at 5 kPa and ϕ_ID_ 410 *μ*m nozzle at 25 kPa (left and right, respectively) and (iv) typical image of the ideal printing conditions. (c) Schematic representation of the skin implant featuring a hypodermis, dual compartment dermis, and epidermis. Inserted micrographs show adipocytes (ADSC), human dermal fibroblasts (HDFs), and human epidermal keratinocytes (hEK) cultured in 2D prior to addition into the construct. (Scale bar represents 200 *μ*m).

The complexity of skin, containing numerous cell types and gradients (both mechanically and chemically), all needs to be replicated to provide optimal integration upon implantation. Suspended manufacture has been shown to provide the high degree of customization required to achieve tissue equivalents.^36^ The technique works via application of a suspending medium known as a fluid gel, which supports the print, while in a low viscosity state, until the construct can undergo full curing [Fig. 1(a)]. The ability to facilitate the full printing process prior to curing is a key feature, not only removing the defined layers (providing a single fully integrated structure), but also broadening the range of potential inks to those with much slower structuring kinetics, typically required by fused deposition processes. To this end, constructs can still be printed at comparable speeds, but the gelation can be undertaken over minutes (as opposed to seconds). Computer aided design (CAD) was used to design a tri-layered construct, which was printed using different bioinks for each layer. The liquid state of the bioinks during processing meant that they remained liquid through the printing process and mixing between the layers at the surface produced structures that did not delaminate.^40^

Recapitulation of the skin's physico-chemical and biological architecture was achieved through careful design of the three layers: epidermal, dermal (both papillary and reticular), and hypodermal as shown by the schematic in Fig. 1(c) and by varying the composition of the matrices. The ECM (extracellular matrix) structure throughout the construct was formed using pectin, a polysaccharide that forms hydrogels upon ionotropic gelation, and is similar to ground substance polysaccharides within native ECM.^41^ A number of studies have demonstrated that this polymer may enhance healing by preventing premature contraction, excessive scarring, and skin distortion.^42^ To further mimic the composition of the skin, collagen was blended with the pectin. The structure was varied through the modification of the ratio between pectin and collagen. In the hypodermal layer, the collagen and pectin were combined at a ratio of 1:1.^43^ The use of a relatively high proportion of pectin enabled the confinement of adipose derived mesenchymal stem cells (ADSC) within the matrix, while providing sufficient structural freedom to enable the migration of surrounding cells into the structure. ADSCs are well known for their role in repair and regeneration, stimulating the dermal fibroblasts, enhancing proliferation and migration, and exhibiting immunoregulatory capabilities, which contribute to the secretion of growth factors, collagens, and fibronectin necessary for good integration of the graft.^18,44,45^ The dermal component of the construct was formed by increasing the collagen content to 2:1. This allowed the higher collagen density associated with the dermis, when compared to the hypodermis.^46^ Changes within the dermal layer to provide replication of both the reticular and papillary layers were achieved through a change in cell density (1.5 × 10^6^ and 3.0 × 10^6^ cells ml^−1^, respectively), again replicating the natural gradients found within native tissue.^47^ Keratinocytes were then seeded atop the dermal compartment to form an epidermis. Much like naturally occurring epidermis, cells were distributed within a sparse ECM compared with ECM-rich connective tissue found within the dermal and hypodermal regions.

One of the key requirements for moving novel 3D printing technologies toward clinical practice is the ability to completely customize each graft/implant. 3D printing has paved the way in bespoke therapies as their ability to converted images/scans of a defect and, through the use of CAD, be able to fabricate an exact match to enhance the regeneration of the tissue in a way that cannot be achieved through a “one size fits all” approach. However, to achieve such complexity, indeed, to print at length scales typical of native tissue microstructures, high print fidelity and resolutions are of the utmost importance. The effect of varying printing parameters such as extruding pressure, flow rate, and nozzle diameter (*ϕ_ID_*) on print resolution has been shown in Fig. 1(b) [the complete set of images can be found in the supplementary material (Fig. s1)]. A linear trend can be seen across all parameters tested (R^2^ = 0.985, 0.952, and 0.926 for constant extrusion pressure, flow rate, and *ϕ_ID_*, respectively), suggesting that as the *ϕ_ID_* and pressure on the system increase, the resolution of the printed structure is lost. However, it can be seen from the insets [Figs. 1(b-iii) and 1(b-iv)] that extremes in the printing parameters can result in loss of fidelity through either under extrusion of the bioink and material failing to be deposited or over extrusion, forcing the material to overcome the confinement of the support material and subsequent seepage/loss of definition. Understanding the printing parameters, thus, provided a set of design rules, which allowed optimal conditions to be determined. Therefore, for the following work, a flow rate of 3.8 *μ*l s^−1^, extrusion pressure of 5 kPa, and *ϕ_ID_* of 410 *μ*m were used to print all further constructs.

The link between printing parameters and mechanical properties of inks has been reported to be directly influenced by the ink's viscosity, nozzle diameter, and flow rate printing pressure.^48^ In particular, the flow properties of an ink play a key role not only in their ability to be extruded, but hysteresis within the materials often dictate print behaviors once deposited on or in the receiving substrate.^49,50^ Furthermore, the viscosity of the ink can result in large stresses when passing through the printing head, often resulting in poor cell compliance and loss of cell viability.^51^

**FIG. 2.**
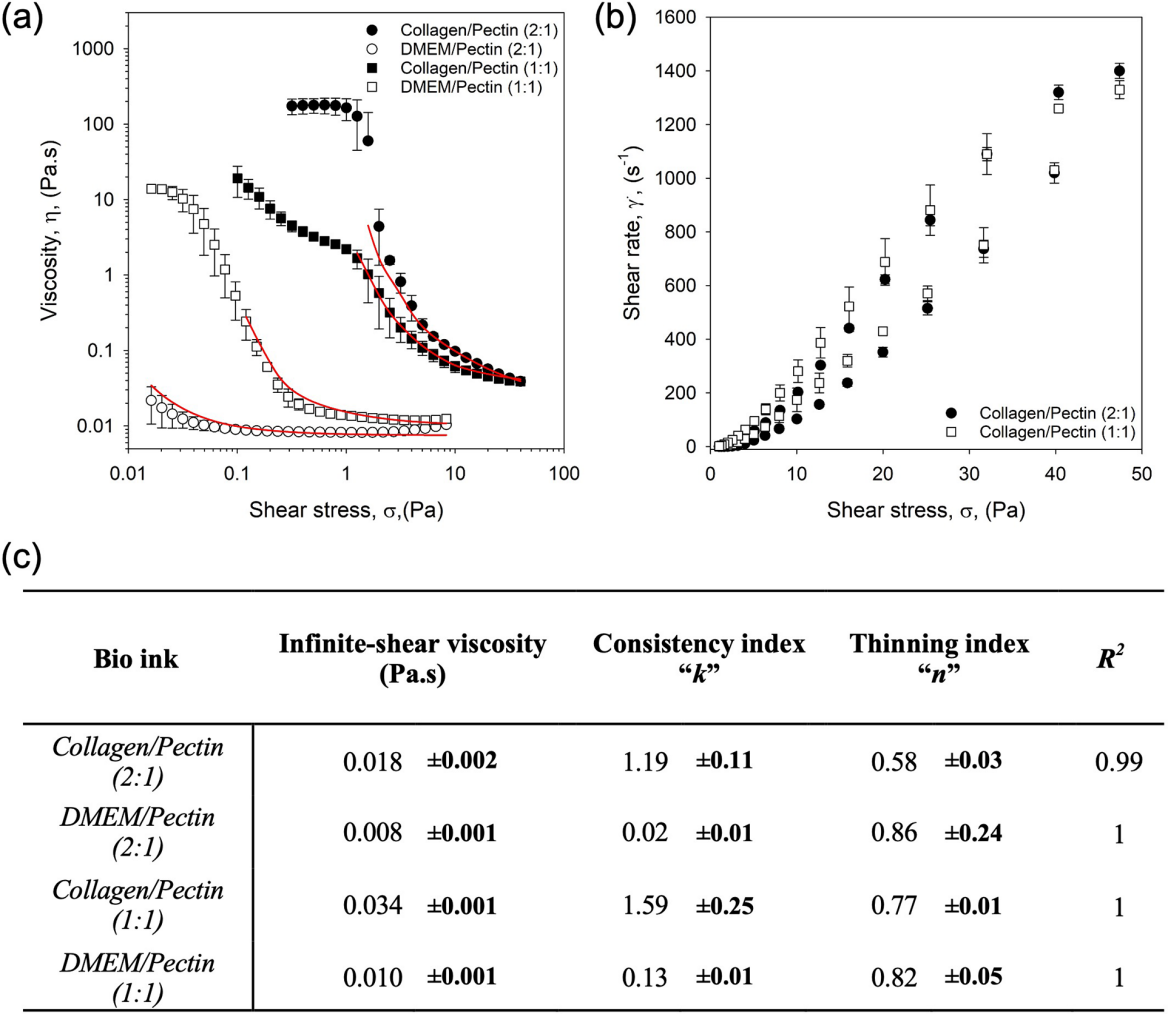
Mechanical characterization of the bioinks: (a) flow profiles for the pectin-based bioinks blended with collagen dissolved in Dulbecco's modified eagle medium (DMEM) (closed markers) and DMEM only (open makers) at a ratio of 2:1 (circles) and 1:1 (squares). Lines demonstrate fit to the Sisko model. (b) Thixotropic analysis of the collagen/pectin bioinks at either a 2:1 blend (circles) or 1:1 blend (squares). (c) Table collating the data obtained for viscometric data fitted to the Sisko model used to determine infinite-shear viscosity.

Time-dependent viscosity data were obtained for pectin-based inks either with or without collagen. In the latter case, DMEM was substituted for the collagen to account for dilution effects (Fig. 2). In all cases, inks demonstrated clear shear-thinning behaviors as a function of increasing shear stress [Fig. 2(a)]. It was observed that when blended with collagen, the viscosity of the bioinks increased across the full sweeps studied. Interestingly, the influence of the collagen on the flow data suggested that it provided a degree of structuring, with the 2:1 blend exhibiting a higher viscosity at lower shear stresses than the 1:1—a trend not observed when the collagen was not present, resulting in dilution of the system and a subsequent loss in viscosity. On further assessment of the flow profiles, it is also possible to see changes in the shape of the curves. In the 1:1 collagen profile, a transient plateauing effect was observed at ca. 0.5 to 1 Pa. Although this transient state cannot be seen in the 2:1 collagen system, it was observed that the first Newtonian plateau extends up to the same point, ca. 1 Pa. It is suggested that such observations are a result of the collagen forming weak structures within the pectin network, producing “yielding-like” behaviors as the weak structures begin to disassemble within the shear field, resulting in ease of flow.^52,53^ Again, similar was observed in the hysteresis data [Fig. 2(b)], where decreasing shear stress sweeps obtained immediately after the first ramp, resulted in an immediate loss of viscosity, likely due to slow ordering kinetics of the collagen.^54^

Viscosity data were fitted to the Sisko model, a typical model used to characterize shear thinning and the second Newtonian plateau, in order to better understand bioink viscosity when undergoing the printing process. Table in Fig. 2(c) shows the fitting data for all systems. Data correlated closely with the model (all R^2^ value >0.99 ± 0.03), providing infinite shear viscosities of 18 ± 2, 34 ± 1, 8 ± 1, and 10 ± 1 mPa s for the 2:1, 1:1 collagen blends and the 2:1, 1:1 DMEM systems, respectively. A combination of Poiseuille's law [Eq. (1)] with an understanding of the relationship between stresses exhibited at the nozzle wall and pressure drop [Eq. (2)] was used to provide Eq. (3), in order to calculate the stress acting upon the cells throughout the print,

$$\Delta p=8.\frac{\eta .l.Q.}{{\pi .r}^{4}},$$ (1)

$$\Delta p=4.\frac{l.\tau_{w}}{D},$$ (2)

$$\tau_{w}=2.\frac{\eta .Q.D}{r^{4}},$$ (3)

where Δ*p* is the pressure drop (Pa), *η* is the infinite shear viscosity (Pa s), *l* is the nozzle length, *Q* is the flow rate of the ink (m^3^ s^−1^), *r* is the nozzle radius (m), *D* is the nozzle diameter (m), and *τ_w_* is the shear stress at the wall (Pa). A range of shear stresses, 14.11 to 59.99 Pa, were determined across the various inks. These relatively low stresses, less than hundreds Pa, exerted within the printing process fall significantly lower than the kPa ranges previously reported to promote loss of cell integrity.^51^ Thus, as a function of the ability to print low viscosity fluids at reasonably slow flowrates, the technique was able to maintain high cell viability post-extrusion, 94.0% ±2.5, and print fidelity, a concept that has historically been difficult to achieve within 3D printing processes.^55^

**FIG. 3.**
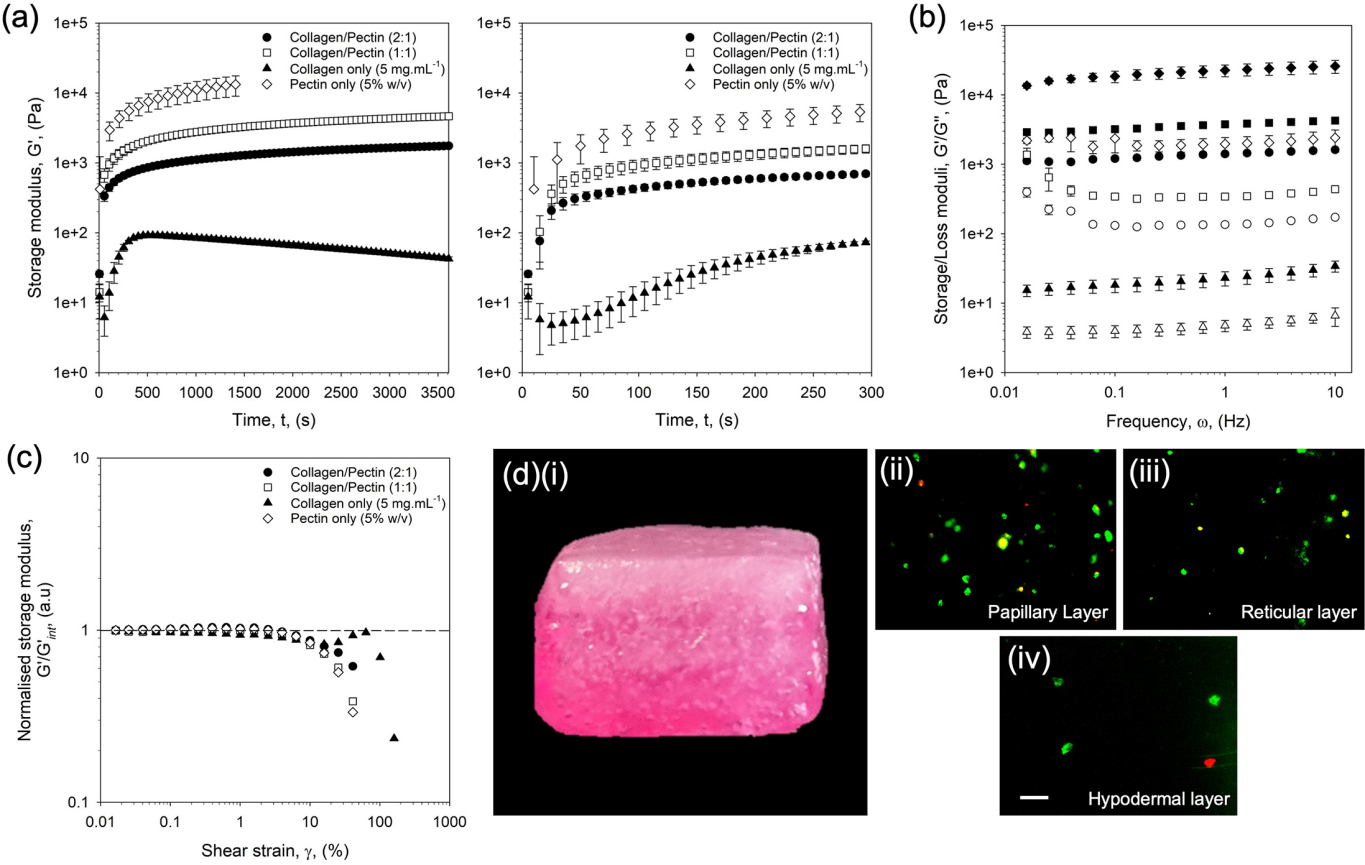
Mechanical characterization of the prints: (a) gelation profiles obtained via a single frequency study (1 Hz, 0.5% strain) for bioink blends and their individual components: 5% pectin only (diamonds), 5 mg ml^−1^ collagen in DMEM (triangles), collagen to pectin blend 1:1 (squares) and collagen to pectin blend 2:1 (circles). Plot on the right focuses on the first 5 min of gelation. (b) Frequency dependent data for each system following gelation: 5% pectin only (diamonds), 5 mg ml^−1^ collagen in DMEM (triangles), collagen to pectin blend 1:1 (squares) and collagen to pectin blend 2:1 (circles). (c) Normalized strain sweeps (at 1 Hz) showing the storage modulus (G′/G′_int_) for each system post-gelation. (d)–(i) Photograph of the whole construct once printed (construct size: 15 × 15 × 9 mm^3^). Increased density of the collagen can be seen through the whitening of the construct toward to the top (purple/pink color is due to indicator within the culture media used). Live dead staining is shown for the (ii) papillary, (iii) reticular, and (iv) hypodermal layers (scale bar depicts 200 *μ*m).

Small deformation rheology was used to probe the gelation and mechanical properties of the discrete layers (Fig. 3). Gelation kinetics, as assessed by the change in storage modulus as a function of time, showed a two-step process, where an initial rapid increase in storage modulus was followed by a much smaller gradient across the timeframe studied [Fig. 3(a)]. Enlargement of the plots to focus on the first 5 min of gelation showed that although systems could be categorized into three groups within the initial gelation step (collagen only, blends, and pectin only—slowest to fastest, respectively), the extent of gelation varied greatly. Indeed, when observing the collagen only profile, a peak modulus was formed at 84 Pa, followed by a slow reduction until the end of the test. It is suggested that such a reduction was a result of the calcium ions within the system (added to simulate the cross-linking agent), slowly diffusing into the gelled structure and intercalating within the triple helix formations, ultimately disrupting the collagenous network.^56^ Such observations were not seen for the blended systems, which continued to increase in storage modulus, likely due to continued diffusion of the cations into the bulk of the matrix, increasing cross-link density. Control over the gelation profiles, although not as critical with suspended manufacturing as other printing technologies (e.g., fused deposition methods which require rapid curing of each layer as it becomes deposited), still plays an important role, providing support for the cells to prevent agglomeration and/or sedimentation. The formulation of the bioink, therefore, is crucial. The pectin in this case provides such control; although slightly slowed through the addition of collagen, it was able to provide the gelled network required within a minute, in comparison to the 5 min required by the collagen only systems.

Interestingly, having undergone gelation, the bulk properties of each layer were dominated by the pectin. Linear rheology was assessed under both dynamic frequency and strain [Figs. 3(b) and 3(c)]. Frequency data [Fig. 3(b)] revealed spectra showing responses typical of hydrogels for all four systems studied (pectin only, 1:1, 2:1, and collagen only).^57,58^ The magnitude of the storage moduli (G′) across the systems highlighted that, whereas with the viscosity data the collagen provided more structure, the presence of increasing collagen to the network resulted in lower values of G′ when measured at 1 Hz (5080 ± 266 and 1900 ± 97 Pa for the 1:1 and 2:1 blends, respectively). Although changes in storage modulus occurred, similar was not observed for values of tan δ (a measure of the ratio of loss to storage moduli), with all pectin containing samples with values within the range of 0.091 ± 0.005 in contrast to 0.208 ± 0.008 for collagen only. This suggests a dilution mechanism, whereby the reduction in storage modulus is a result of a less dense pectin network, as opposed to the collagen physically blocking the cross-linking moieties. A similar inference can be drawn from the frequency sweep profiles, again similar across all pectin containing systems, suggesting comparable polymer networks throughout the samples. Strain sweeps [Fig. 3(c)] seem to confirm this, with data superimposable, showing constant linear viscoelastic regions and yielding behaviors across all samples. It is important to note that out of the linear region, a degree of strain hardening was observed for the collagen only systems, not observed for the 1:1 blend. However, as more collagen was added to the system, in the 2:1 hydrogel, small deviations in the yielding rate were observed, shifting it toward that of the collagen only.

The rheological data suggest a complexity within the microstructure, whereby the continuous network is provided by the pectin, allowing the collagen to amass within the interstitial area. This provides a method of creating a dual network, with the opportunity for cells to remodel the collagen without compromising the integrity of the overall construct. Again, such structures are akin to the native tissue, where the ground substance creates the sub-structure for collagenous matrix to be modeled within.^59,60^ Furthermore, the ratio of collagen to pectin provided a means to control the hydrogel stiffness throughout the construct. To this end, it was possible to show various layers with moduli comparable to actual skin mechanics,^61^ thus providing not only the potential for bespoke microenvironments critical for maintaining cell viability (77.9% ±3.3 post-gelation and culture) [Fig. 3(d)], but also maintaining a platform to drive cell response, as described in literature.^62–64^

### *Ex vivo* implantation of the tri-layered skin construct

**FIG. 4.**
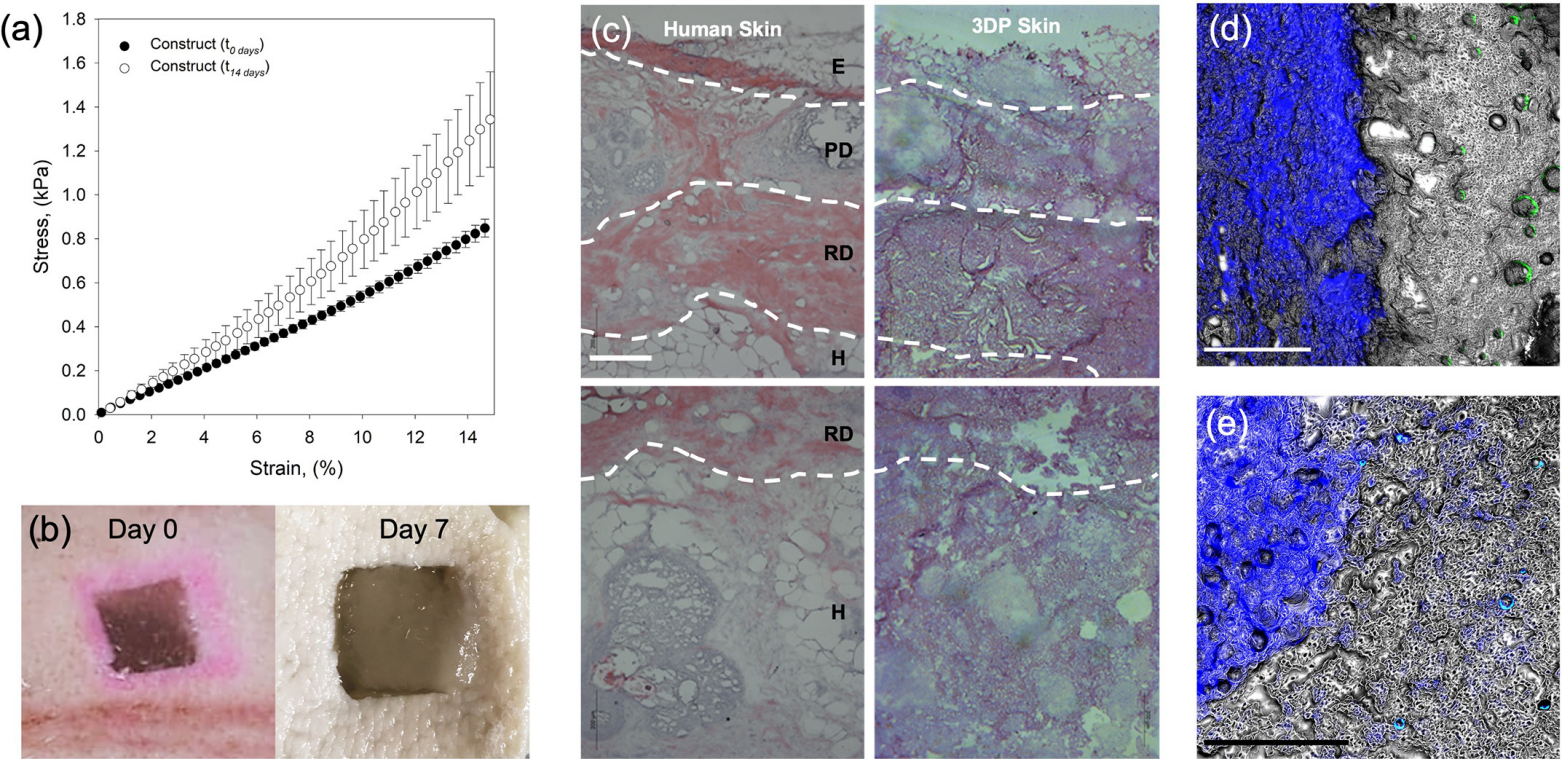
Integration of the bioprinted skin within a simulated wound. (a) Mechanical analysis (compression) of the skin construct post-printing and after 14 days of incubation. (b) Images of the skin construct directly after implantation within the defect and after seven days of culture. (c) Histological characterization of: (right) human skin highlighting the epidermis “E,” papillary dermis “PD,” reticular dermis “RD,” and hypodermis “H” using hematoxylin and eosin staining; (left) 3D printed skin, after 21 days of culture (scale bar denotes 200 *μ*m). (d) Fluorescence microscopy of the interface between the porcine tissue (blue) and implant (gray) focused on the papillary and reticular layers highlighting the formation of HDF clusters (green) after seven days of culture (Scale bar denotes 500 *μ*m). (e) Fluorescence microscopy focused on the interface within the hypodermis layer after seven days of culture. Infiltration of the surrounding porcine tissue (blue) can be seen within the implanted construct (scale bar denotes 500 *μ*m).

Application of the printed structure as an implant was assessed *ex vivo*, using a simulated porcine wound. Although the simulated wound has not been previously proven as a model for chronic wounds, not mimicking the typical harsh environments often observed, it provided the means to study the integration of the implant within the defect site alongside changes in the implant's architecture. Mechanical behavior of the bulk construct was determined directly post-curing and compared to that of a construct having undergone 14 days of incubation [Fig. 4(a)]. Compression data revealed ageing of the materials over the incubation time, with Young's modulus increasing from 4726 ± 172 to 6966 ± 1172 Pa. Such observations are likely due to continued collagen fibrillogenesis and re-arrangement, reinforcing the network and resulting in a stiffer matrix. Visual inspection of the constructs also seems to correlate with this, with implants becoming increasingly turbid [Fig. 4(b)] throughout the incubation. Indeed, the dynamic behavior facilitates an environment more aligned with native mechanical properties and changes in collagen microstructures, resembling the normal healing processes and regeneration of the tissue.

The resemblance of the implant to human skin was evaluated after 21 days of culture, using histological staining [hematoxylin and eosin (H&E)] [Fig. 4(c)]. The skin can be easily segmented into three main areas: the epidermis, dermis, and adipose tissue (comprising of the hypodermis and subcutaneous tissue), each with an inherent structure. Although the presence of calcium within the pectin allowed for binding of the hematoxylin, convoluting spatial distribution of eosin (collagen) within the implant, similarities with the human skin could still be seen. In particular, a clear distinction between the dermis and hypodermis was observed, characterized by the density of the matrix within each layer. The role in which the cellular components played was also clear, providing the notable vacuolated structures within the hypodermis, indicative of the native architecture. Moreover, the resemblance between the skin and implant, not only mechanically (as previously demonstrated), but structurally, compositionally, and on a cellular level, provides similar platforms in which the two can integrate.

Integration of the implant after seven days of culture was studied though visualization of the tissue–implant interface [Figs. 4(d) and 4(e)]. A clear distinction between tissue and implant could be seen within the dermis [Fig. 4(d)]; however, a seamless continuity between tissue and construct matrix (small fractures are believed to be an artifact of the sectioning process) suggested full integration of the implant. As observed through the H&E staining for the adipocytes within the hypodermis, cellular structures could also be seen within the dermis: with fibroblasts (labeled using fluorescent trackers) creating initial cluster formations, spread throughout the implant.^65^ Unfortunately, cell migration into the surrounding tissue could not be seen, potentially masked by the tissue's autoflorescence. Moreover, even at seven days of culture, it was possible to see some hypodermal tissue within the implant structure [Fig. 4(e)]. Mobilization of the tissue at such an early timepoint is very promising, especially within deep wound repair, where recent studies have shown healing to be achieved through movement of the fascia upward toward the surface.^12^

The results obtained within the *ex vivo* study showed that by providing an implant with correct compositional, mechanical, and cellular cues, it was possible to integrate with the surrounding tissue, with the production of key architectures and microstructures found in native skin. Although currently in its early stages, mobilization of the tissue surrounding the grafts suggests the potential to facilitate tissue repair without a scar. Indeed, the potential to promote mobilization of the fascia could provide a platform, when in a biological setting (endogenous cytokines, sentry fibroblasts etc.), to drive the formation of secondary structures such as vessels or niches. However, further biological characterization is required to substantiate such hypotheses.

## CONCLUSIONS

A skin equivalent complete with a tri-culture of cells distributed throughout hypodermal, dermal, and epidermal regions was successfully designed and fabricated. Suspended layer additive manufacture provided the ability to print bespoke constructs, where high precision and resolutions made it possible to create implants with compositional, mechanical, and cellular replication of native skin. Moreover, the ability to print low-viscosity inks, at relatively slow speeds, facilitated minimal stresses exerted on the cells throughout processing. As such, continuous constructs were formed demonstrating mechanical gradients throughout. Rheological characterization was used to probe the microstructure of the implants. It was observed that throughout each layer, the structure was dominated by the pectin, with the collagen remaining in the interstitial voids. This provided a mimetic for the native skin, where ground substance proteoglycans provide the support for the collagen. Scaffolds also demonstrated their potential for integration within an *ex vivo* simulated skin wound. Here, the ability to recapitulate both the skin's microstructures and cellular environments facilitated the ability to mimic native architectures under culture. To this end, similarity between both implant and the tissue ultimately led to high levels of integration. Furthermore, it was observed that mobilization of the hypodermal tissue was achieved within seven days of culture, in line with known mechanisms for deep wound healing. Therefore, these implants have the potential to mimic skin, providing an implant that is much closer to that of human skin, which could be used to enhance wound repair.

## METHODS AND MATERIALS

### Materials

Low methoxy (LM) pectin was purchased from CP Kelco, UK; collagen (PureCol EZ Gel, Advanced BioMatrix), CaCl_2_·2H_2_O, DMEM, HEPES, adipogenic media, dispase II solution, trypsin, penicillin/streptomycin, and paraffin were all purchased from Sigma-Aldrich, UK; keratinocyte growth medium was purchased from KGM, Lonza, UK; and calcein AM (acetoxymethyl ester) and propidium iodide were purchased from Fisher Scientific, UK.

### Preparation of printing media

Stock suspending medium [0.5% (w/v)] was prepared through the addition of agarose to de-ionized in a Schott bottle. Once dispersed, a magnetic stirrer bar was added to the suspension and autoclaved (121 °C, 15 min). On removal from the autoclave, the solution was constantly mixed (700 rpm) throughout cooling to 25 °C. Stocks were kept sealed to maintain sterility at 4 °C until further use.

Stock pectin solutions [5% (w/v)] were initially prepared by dispersing dry powder in de-ionized water under constant agitation. Once dispersed, the colloidal suspension was autoclaved (121 °C, 15 min) so form sterile working stocks and allowed to cool (25 °C). Where appropriate, the stock was further mixed (2:1 or 1:1) with either collagen (5 mg ml^−1^ in DMEM) or standard DMEM in a class II biological cabinet to provide working bioinks.

### Isolation of human cells from skin

Human skin tissue complete with subcutaneous adipose was purchased under the French (law L. 1245 CSP) from Genoskin (Fr) from an elective human abdominoplasty, as “a product and element of body taken during a surgical procedure and used for scientific research,” with the patients consent—no further ethics approval was required. Samples were shipped at 4 °C in transportation culture media within 4 h of excision. Dermo-epidermal tissue was sectioned and submerged in dispase II solution at 4 °C overnight followed by separating the dermal and epidermal counterparts.

Isolation of the dermal fibroblasts (HDF) was achieved by incubation of the dermis in collagenase D solution overnight, followed by trypsin treatment and mechanical agitation using a scalpel. Cells were centrifuged, re-suspended in basal media [Dulbecco's modified eagle medium (DMEM) supplemented with FBS (fetal bovine serum) (10%), HEPES (2.5%), and penicillin/streptomycin (1%)] and filtered through a cell strainer (100 *μ*m) before expanding in a T25 flask.

Isolation of epidermal keratinocytes (hEK) was achieved by trypsin treatment followed by mechanical mincing. Keratinocyte growth medium (KGM, Lonza, UK) was used to quench enzymatic action followed by centrifugation, re-suspension, and filtration (70 *μ*m cell strainer). Cells were then plated onto mitomycin-C treated 3T3-J2 murine fibroblast feeder layers.

Stromal vascular fraction (SVF) was isolated from adipose tissue by mechanical mincing, followed by sieving (dissociation sieve size 100 mesh) and incubating in collagenase type I. Media was used to quench the reaction and samples sequentially filtered through 100 and 40 *μ*m filters. Solutions were centrifuged, and the lipid, mature adipocyte, and aqueous layers discarded. The pellet was suspended in erythrocyte lysis buffer followed by further centrifugation prior to plating in basal media. Extracted SVF was sub-cultured as per standard trypsinization protocols for 21 days to produce an ADSC population. ADSCs cultured for 21 days were washed in PBS (phosphate buffered saline) and adipogenic differentiation medium (basal media supplemented with 500 *μ*M isobutyl-methylxanthine (IBMX), 50 *μ*M indomethacin, and 1 *μ*M dexamethasone) added. Cultures were maintained for a further 21 days.

### Determination of ADSC multipotency

To confirm the mesenchymal status of the SVF cells culture under osteogenic or adipogenic conditions was undertaken, followed by staining for typical markers (lipid/calcium mineral) and flow cytometry. In brief, SVF cells were cultured in either adipogenic media [basal media supplemented with 500 *μ*M isobutyl-methylxanthine (IBMX), 50 *μ*M indomethacin, and 1 *μ*M dexamethasone] or osteogenic media (basal media supplemented with 10 mM β-glycerophosphate, 200 *μ*M ascorbic acid, and 0.1 *μ*M dexamethasone) for 21 days. Following culture, cells were fixed in 10% formalin. Cells were then stained with either Oil Red O solution (60%) or Alizarin Red S solution (2%) and visualized under an inverted microscope (Fig. s2). Phenotypic analysis was undertaken using flow cytometry (Millipore Guava Easycyte Flow Cytometer, Merck Millipore, UK) in order to confirm the heterogeneity of freshly isolated SVF populations at week 0 and the homogeneity at 21 days. Cells were trypsinized and re-suspended in PBS. An antibody cocktail (anti-human CD34 PE/CD45 FITC, BD Biosciences) was added to the suspension and incubated at room temperature. Nonspecific binding (mouse IgG1 PE and mouse IgG1 FITC, BD Biosciences) was determined through addition of a control antibody to separate samples, alongside nontreated cells for cell counting. Mean fluorescence intensity was adjusted for nonspecific binding (δMFI—mean fluorescence intensity) (Fig. s2).

### Optimization of the printing process

Optimum printing parameters were obtained by systematically altering parameters such as nozzle diameter (*ϕ_ID_*) and printing pressure while maintaining a constant printing speed of 10 mm s^−1^ at room temperature, in brief: An INKREDIBLE™ 3D bioprinter (Cellink, Sweden) was used to print bioinks into the suspending media at constant pressure, flow rate, and nozzle diameter to assess resultant print resolution. The bioink was died blue and single layer rings (15 mm diameter) printed at a pressure ranging between 5 and 25 kPa (5 kPa increments) through a *ϕ_ID_* of 150 *μ*m. The process was repeated at constant pressure (24 kPa) varying *ϕ_ID_* (150, 200, 250, 410, and 610 *μ*m). The width of the extrudate was then measured using digital calipers and reported. Optimal printing conditions were obtained to be: flow rate—3.8 *μ*l s^−1^, pressure—5 kPa, and *ϕ_ID_* 410 *μ*m.

### Fabrication of implant constructs

Blends of collagen to pectin (2:1 and 1:1) were used to re-suspend pellets of cells resulting in three final bioinks: 2:1 blend containing 3.0 × 10^6^ cells ml^−1^ (HDFs—passage 7); 2:1 blend containing 1.5 × 10^6^ cells ml^−1^ (HDFs—passage 7); and a 1:1 blend containing 5 × 10^5^ cells ml^−1^ (ADCSs—passage 5) for the papillary, reticular, and hypodermal layers, respectively. The bioprinter was aligned and calibrated to the optimized printing parameters followed by placement of the petri dish containing the agarose fluid bed upon the z-stage. The G-code for the construct was uploaded to the printing software to print a tri-layer consisting of the following dimensions: hypodermis layer (15 × 15 × 7 mm^3^), reticular layer (15 × 15 × 1 mm^3^), and papillary layer (15 × 15 × 1 mm^3^). Inks were automatically switched upon each successive layer, resulting in constructs printing in ca. 13 mins. Following fabrication, constructs were gelled using CaCl_2_ (200 mM) through injection to the local area. Immediately following the addition of cross-linking agent, adipogenic media [basal media supplemented with 500 *μ*M isobutyl-methylxanthine (IBMX), 50 *μ*M indomethacin, and 1 *μ*M dexamethasone] was added to the suspension bath and constructs incubated (37 °C, 5% CO_2_, and 95% air) to allow the collagen to undergo fibrillogenesis. The following day, constructs were removed from the printing bath and subsequently cultured in adipogenic media for the following 14 days. Keratinocytes (2 × 10^6^—passage 6) were then seeded on the top of the construct and cultured for a further seven days; once confluent, the top of the construct was raised to the air–liquid interface to create an epidermal layer.

### Mechanical characterization

All rheological characterizations were conducted using a Kinexus Ultra+ rotational rheometer (Netzsch, DE).

Dynamic viscometric measurements were obtained using a cone and plate geometry (4°, 40 mm diameter) at 25 °C. Shear stress ramps were conducted between 0.01 and 100 Pa (upper limits were determined by the sample being tested) over a 3 min ramp time. A second sweep was performed immediately following the first ramp at decreasing shear stresses to probe thixotropic effects.

Small deformation rheology was used to characterize bioink gelation and subsequent viscoelastic properties. A serrated parallel plate (40 mm diameter, 1 mm gap height) was used to conduct the studies at 37 °C. A second outer ring was applied to create a well where the addition of the cross-linking agent [aqueous CaCl_2_ (200 mM)] was added after the test had been started. Gelation was studies performed using a single frequency test (1 Hz, 0.5% strain) over 60 min. Before removal of the gel, *in situ* frequency sweeps were obtained at constant strain (0.5%) between 0.01 and 10 Hz, followed by an amplitude sweep (1 Hz, 0.01% to 500% strain).

Compressional rheology was undertaken using a dynamic mechanical analyzer (Bose, US), using a 0.25 N load cell. Tests were conducted under uniaxial compression at a rate of 0.01 mm s^−1^ to a maximum strain of 15%.

### Live/dead cell viability

3D printed skin equivalents were sectioned and stained with calcein AM (staining live cells green) and propidium iodide (PI—staining dead cells red) reagents (Fisher Scientific, UK) for the visualization of suspended cells. Constructs were then visualized using a fluorescent microscope (EVOS FLoid Cell Imaging Station, ThermoFisher Scientific, UK).

### *Ex vivo* implant integration study

Freshly slaughtered porcine skin was sourced direct from the abattoir and washed in absolute ethanol before forming a simulated wound throughout the epidermal, dermal, and hypodermal layers using a scalpel. HDFs were labeled using a Q-Tracker cell labeling kit (kit 488), as per the kit's instructions prior to addition to the bioink. A skin equivalent was then 3D printed to fit the dimensions of the porcine wound model followed by solidification and placement of the implant within the wound cavity. Samples were then cultured for up to 21 days before fixing, embedding and sectioning.

### Embedding of implants

3D printed skin equivalents and human skin samples were embedded in either paraffin or OCT (optimal cutting temperature compound). All samples were fixed using formalin (10%). Paraffin embedded samples were then dehydrated using a series of washes with increasing concentration of ethanol. Samples were then placed in xylene prior to embedding (Leica EG 1150C Tissue Embedder, Leica Biosystems, UK). OCT samples, post-fixing, were subsequently treated with increasing concentrations of sucrose solution as a cryoprotectant and frozen in OCT.

### Hematoxylin and eosin (H&E) staining

Paraffin embedded samples were sectioned to 7 *μ*m thickness and mounted onto adhesion coated slides. Slides were subjected to immersion within xylene followed by a series rehydration within graded ethanol (absolute, 70%, and 30%). Slides were then rinsed under running tap water between treatments first with hematoxylin followed by counterstaining with eosin. Slides were dehydrated in a series of ethanol (30%, 70%, and absolute) and submerged in xylene before the addition of mounting solution and a coverslip prior to analysis under a Leica CTR 6500 confocal microscope (Leica microsystems, UK).

## SUPPLEMENTARY MATERIAL

See the supplementary material for the complete panel of print resolutions used to determine optimal printing parameters and data showing ADSC isolation and multipotency.

## Data Availability

The data that support the findings of this study are available from the corresponding authors upon reasonable request.
